# Asymmetric Distribution of GFAP in Glioma Multipotent Cells

**DOI:** 10.1371/journal.pone.0151274

**Published:** 2016-03-08

**Authors:** Pierre-Olivier Guichet, Sophie Guelfi, Chantal Ripoll, Marisa Teigell, Jean-Charles Sabourin, Luc Bauchet, Valérie Rigau, Bernard Rothhut, Jean-Philippe Hugnot

**Affiliations:** 1 INSERM U1051, Institut des Neurosciences de Montpellier, Hôpital St Eloi, 80 avenue Augustin Fliche, 34091 Montpellier Cedex 05, France; 2 CHU Montpellier, Hopital Gui de Chaulliac, 80, avenue Augustin Fliche, 34295 Montpellier, France; 3 Université Montpellier, Place Eugène Bataillon, 34095 Montpellier Cedex 5, France; National Cancer Institute, UNITED STATES

## Abstract

Asymmetric division (AD) is a fundamental mechanism whereby unequal inheritance of various cellular compounds during mitosis generates unequal fate in the two daughter cells. Unequal repartitions of transcription factors, receptors as well as mRNA have been abundantly described in AD. In contrast, the involvement of intermediate filaments in this process is still largely unknown. AD occurs in stem cells during development but was also recently observed in cancer stem cells. Here, we demonstrate the asymmetric distribution of the main astrocytic intermediate filament, namely the glial fibrillary acid protein (GFAP), in mitotic glioma multipotent cells isolated from glioblastoma (GBM), the most frequent type of brain tumor. Unequal mitotic repartition of GFAP was also observed in mice non-tumoral neural stem cells indicating that this process occurs across species and is not restricted to cancerous cells. Immunofluorescence and videomicroscopy were used to capture these rare and transient events. Considering the role of intermediate filaments in cytoplasm organization and cell signaling, we propose that asymmetric distribution of GFAP could possibly participate in the regulation of normal and cancerous neural stem cell fate.

## Introduction

Asymmetric distribution of molecules during division is a fundamental mechanism which has a major impact on the formation of cell diversity and final size of the organs [1–3]. Unequal repartition of proteins, such as transcription factors and growth factor receptors, but also other cellular constituents such as mRNA or even organelles, will generate unequal cell fates from genetically identical daughter cells [4–7]. In accordance with their central role in development, several proteins involved in the Notch and Wnt pathways have been described to be asymmetrically distributed during division [8, 9].

Close links between asymmetric division and cancer have also been established [10–12]. Particularly in Drosophila, mutation of genes involved in asymmetric division can result in uncontrolled proliferation and cancer [13]. In mammals, reduction of asymmetric repartition of the proteoglycan NG2 during division of oligodendrocyte progenitor cells correlates with formation of brain tumors [14]. Defect in asymmetric division may also contribute to the formation and persistence of cancer stem cells [15].These cells, which have been described in many types of tumors, are more resistant to conventional treatments and are thought to be at the origin of tumor recurrence [16, 17]. They express specific markers such as CD133 which can be asymmetrically distributed during division [18].

A category of protein which has been given little attention in the asymmetric division process is intermediate filaments. This large family of cytoskeleton proteins perform many cellular functions beyond their well-documented role for the regulation of cell shape and intracellular organization [19, 20]. These are for instance guidance of signalling factors and mitochondria motility [21, 22]. More recently, it was demonstrated that the intermediate filament vimentin mediates the asymmetric partitioning of damaged, misfold and aggregated proteins in JUNQ inclusion bodies in mammalian cells which provide new biological insight into the role of intermediate filaments in cell rejuvenation [23].

In this article, we focused on the GFAP intermediate filament which is expressed in mature astrocytes in the nervous system. However, it is now well established that GFAP can also label immature oligodendroglia as well as adult neural stem cells [24–27]. GFAP is also highly expressed in astrocytoma such as glioblastomas, the most frequent and devastating type of brain tumors [28]. These tumors contain a subpopulation of cancer stem cells which can be propagated as spheres in defined media [29–31]. Here we explored the expression of GFAP in glioma spheres derived from GBM patients. Using immunofluorescence (IF) and videomicroscopy, we demonstrated that GFAP can be asymmetrically distributed during division. Similar observations were made in non tumoral neural stem cell cultures. This unequal repartition of GFAP possibly plays a role in the asymmetric division process and cell fate regulation.

## Materials and Methods

### Cell culture and Isolation of clonal multipotent cell line mGb4

Isolation of mGb4 clonal cell line was performed by single cell seeding of Gb4 GBM primary culture [32] in 96-well plates with an Aria cytometer (BD). Adult spinal cord neural stem cells were obtained from adult mice (CB1, Charles River, France) and cultured as described in [33, 34]. Medium consisted of DMEM/F12 1:1 (Invitrogen), N2 and B27 supplements (Invitrogen), 2mM glutamine (Invitrogen), 0.6% glucose (Sigma), 20 μg/ml bovine insulin (Sigma) supplemented by 2 μg/ml Heparin (Sigma), 20ng/ml EGF (Peprotech) and 10ng/ml FGF2 (Peprotech). For differentiation and upregulation of GFAP, mGb4 cells were plated adherently for 5 days on poly-D-lysine (25 μg/mL) and laminin (2 μg/cm^2^)-coated glass coverslips in medium containing 0.5% fetal bovine serum instead of EGF and FGF2 growth factors. Isolation of Gb5 cells were described in [32] and Gb21 cells were isolated using the same procedure. Information for Gb4, Gb5 and Gb21 patients are provided in S1 Table. Intracranial transplantation of mGb4 cells was performed as described in [32]. Mice were handled following the guidelines of the Animal Care and Use Committee of the Institut National de la Santé et de la Recherche Médicale (INSERM) who approved this study in accordance with the European Council directive (2010/63/UE) for the protection and use of vertebrate animals (Permit Number: CEEA-LR-11038). All transplantations were performed under isoflurane gas (1.5%) and every effort was made to minimize the number and suffering of animals. Three months after transplantation, mice were euthanatized by intraperitoneal injection of sodium pentobarbital (100 mg/kg).

### Transfection

Human GFAPα cDNA cloned into pEGFP-C2 (hGFAPα-EGFP-C2) or pEGFP-N3 (hGFAPα-EGFP-N3) and human GFAPε cDNA cloned into pEGFP-N3 (hGFAPε-EGFP-N3) were kindly provided by Dr. D. Pham Dinh (Paris, France). These constructions were transfected in mGb4 cells using an Amaxa apparatus and neural stem cell kit (Lonza). For videomicroscopy, the H2B-mCherry plasmid (Addgene, 20972) was co transfected to visualize the nuclei in the red channel. Transfection with pEGFP-N3 (pCMV-EGFP-N3) plasmid (Clontech) was used as control. For visualization of Golgi apparatus, mGb4 cells were transfected with a pDsRed-Monomer-Golgi (Clontech). After 48h, cells were processed for time-lapse confocal microscopy or fixed with 4% paraformaldehyde 20 min RT, for IF.

### Immunofluorescence and imaging

IF was performed as previously described in [32]. Briefly, cells were fixed with 4% PFA for 20 min at room temperature, washed twice with PBS and permeabilized with a blocking solution containing 5% donkey serum 0.01% triton X-100 diluted in PBS. Then, cells were incubated overnight with the appropriate primary antibody (S2 Table). Immunoreactivity was visualized using Alexa-488 or Alexa-594 donkey anti-mouse, anti-rabbit or anti-chicken secondary antibodies at a dilution 1:1000 (Jackson ImmunoResearch). Nuclei were counterstained with Hoechst 33242 (blue on images). Glass coverslips were mounted using fluorescence Mounting Medium (Dako, S3023). Images were captured with an AxioImager Z1/Apoptome microscope (Zeiss).

### Flow cytometry

FACS was performed on mGb4 and Gb21 living cells stained directly by PE-conjugated CD44 (5 μg/ml) or indirectly by CD133 (5 μg/ml) or CD15 (5 μg/ml) followed by anti-mouse Alexa A488 (Jackson Lab) diluted in PBS 0.5% BSA 2 mM EDTA 0.1% Azide. Control cells were incubated with control isotype antibodies in the same buffer. Cells were analyzed on a Galios flow cytometer (Beckman Coulter) and analysis was performed on Kaluza (Beckman Coulter).

### Time-lapse confocal microscopy

mGb4 cells were grown in 35 mm glass dish coated with poly-D-lysine/laminin and imaged on LSM5 live duo inverted Microscope (Zeiss) equipped for time-lapse microscopy with CCD camera, temperature controller (37°C) and CO2 (5%) incubation chamber. Cell divisions were imaged as stack images at 0.5–1 μm z-spacing with an objective EC Plan-Neofluar 40x/1.30 Oil DIC M27 (Zeiss). After acquisition, images were analysed using LSM5 images browser (Zeiss). For best viewing of proteins distribution, all z-planes were merged in one single plane projection. Raw data have been deposited on the public repository Figshare and are available at this link https://figshare.com/s/b332c725ae0695770cfb.

### Statistical analysis of asymmetry

GFAP asymmetric repartition events were scored in mitotic cells engaged in the late mitosis steps i.e. in anaphase or telophase with two separate chromatid sets but still sharing a common cytoplasm. Z acquisitions were systemically performed to ascertain that GFAP asymmetry was present in all or most cell planes to avoid artefact with cells displaying a 3D peculiar shape. To quantify the asymmetry in IF and videomicroscopy experiments, we determined the percent (%) deviation previously described in [18] using this formula: (F1-F2)/ (F1+F2) x 100 where F1 and F2 represent the fluorescence of each cells (using CTCF, i.e. integrated density–(area of selected cell X mean fluorescence of background readings) obtained with imageJ software). Fluorescence of β-tubulin (which is equally expressed in both daughter cells) was used to determine the asymmetry cell division cutoff. The % of deviation for β-tubulin (quantified for 20 dividing cells) was 5.834% and the deviation standard was 4.358. We set the cutoff to be greater than the 99% confidence interval of β-tubulin. For a bell-shaped distribution, this calculates to the mean difference +/- 2.845 x standard deviation (t-test table) which is 18.23% for the upper limit. This led us to set the asymmetry cutoff to be greater than a 20% difference between sibling cells.

## Results

### Isolation of a clonal multipotent GBM cell line

**Fig 1:**
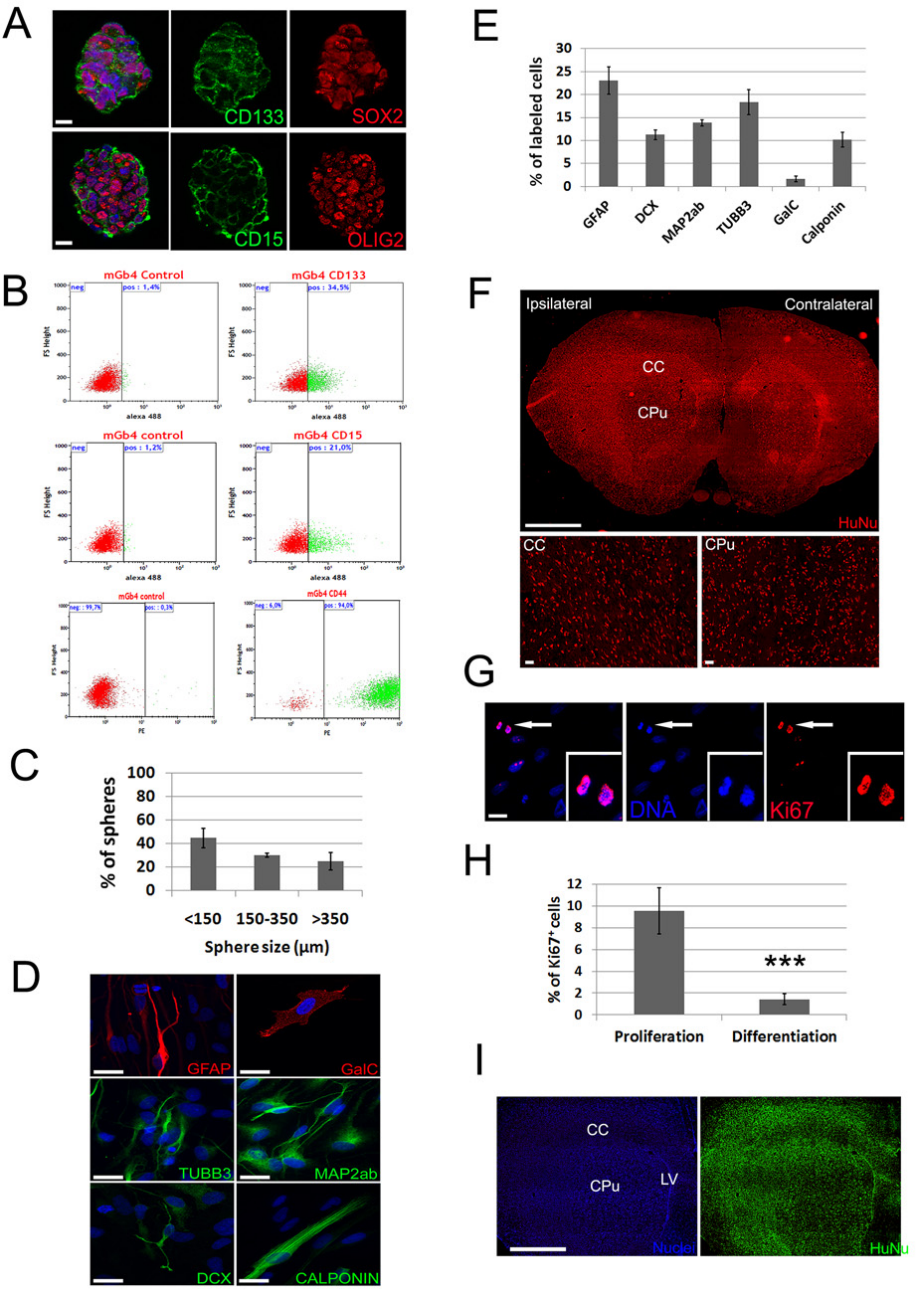
Characterization of mGb4 clonal cell line. **(A)** IF for the indicated markers performed on mGb4 neurospheres. These cultures express CD133, CD15, OLIG2 and SOX2. **(B)** FACS analysis for CD133, CD15 and CD44 expression in mGb4 cells. **(C)** Quantification of clonal neurosphere formation in 96 well plate one month after seeding as a function of their size at three different passages. **(D)** mGb4 cell multipotency analysis. Examples of cells stained for the indicated markers, observed 5 days after differentiation. **(E)** Quantification of experiment presented in **(D)**. **(F)** Detection of mGb4 transplanted cells (red dots) by IF for Human Nuclei antigen (HuNu) 3 months after transplantation. Note the profuse invasion of the ipsilateral hemisphere (right-hand hemisphere), corpus callosum (CC), caudate putamen (CPu) and contralateral hemisphere (left-hand hemisphere). Insets show high density of HuNu^+^ cells in the corpus callosum and caudate-putamen regions **(G)** Dividing mGb4 cells (arrow) grown in differentiating condition can be observed by Ki67 staining (right-hand image) and highly-condensed chromatin visualized with Hoechst staining (middle image). Inset shows high magnification of a Ki67 staining associated with chromosomes in mGb4 mitotic cell. **(H)** Quantification of KI67^high^ mitotic mGb4 cells in proliferating or after 5 days of differentiation (n = 7 fields, Mann-Whitney rank sum test, *** (p<0.001). **(I)** Examples of tumoral cells (visualized by HuNu staining, green) detected in the CC and CPu of a mice transplanted with 100 000 mGb4 cells which had been differentiated for 5 days. Scale bar = 10 μm (A, D, G), = 2.5 mm (F, upper image; I), = 20 μm (F, lower images).

We previously isolated and characterized several cultures derived from GBM patients [32]. These cells grow as neurospheres in defined media and meet criteria for classifying them as multipotent glioma cells. These cultures are however heterogeneous and contains a mixture of cells carrying various mutations, different proliferation rates and differentiation potentials. In order to restrict our study to clonally-expanded, highly-proliferative and multipotent GBM cells, we isolated a cell line by single-cell seeding in 96 wells plate. Characterization of the resulting cell line (mGb4) by IF and FACS revealed that cells express CD15, CD133, CD44 stem cell markers together with SOX2 and OLIG2, two transcription factors associated with the maintenance of multipotency and proliferation of glioma multipotent cells (Fig 1A and 1B). Clonogenicity analysis performed at three different passages in 96 wells plate indicated that approximately 20% of mGb4 cells were able to form new large (>350 μm) and passageable neurospheres (Fig 1C). Multipotentiality of mGb4 cells was tested by plating them on adherent substrate without growth factors. In this condition, mGb4 cells generated neuronal (DCX^+^, MAP2ab^+^, TUBB3^+^), glial (GFAP^+^, GalC^+^) and mesenchymal-like (Calponin^+^) cells (Fig 1D and 1E). Finally, intracranial transplantation of mGb4 cells in nude mice generated infiltrative tumors (10/10 transplanted mice) after 3 months, thus confirming the tumoral properties of these cells (Fig 1F). Taken together, these results validate the use of mGb4 clonal cells as a cellular model for GBM multipotent cells.

Interestingly, we noted that after growth factors removal (i.e. differentiation condition), mGb4 cells still proliferate, albeit at a lower rate, as evidenced by the presence of Ki67^+^ cells and mitotic cells (Fig 1G and 1H). Moreover, these cells also generate tumors after intracranial transplantation (Fig 1I) suggesting that they have not reached terminal differentiation but rather remain immature and blocked in their final differentiation process probably as the result of mutations interfering with the cell cycle exist [35].

### Asymmetric distribution of GFAP during mitosis in GBM and mNSC cultures

**Fig 2:**
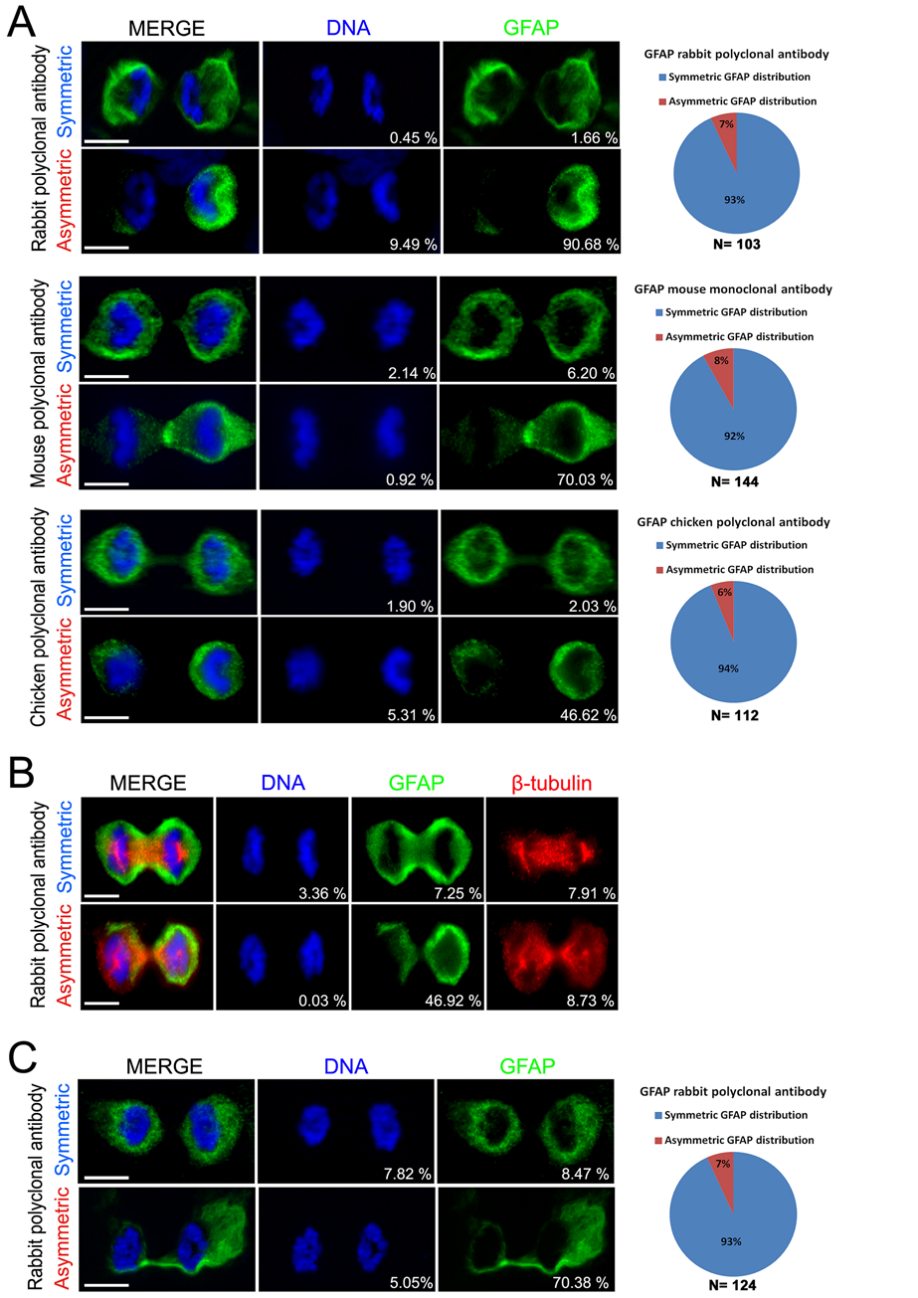
Symmetric and Asymmetric distribution of GFAP in mGb4 and mNSC observed by IF. **(A)** Examples of symmetric and asymmetric distribution of GFAP during mitosis in mGb4 cells detected using a rabbit polyclonal antibody (Z0034, upper panel), mouse monoclonal antibody (G3893, middle panel) or chicken polyclonal antibody (AB4674, lower panel). **(B)** Examples of asymmetric and symmetric distribution of GFAP in mitotic cells stained for spindle apparatus (β-tubulin in red). **(C)** Examples of symmetric and asymmetric distribution of GFAP in mitotic spinal cord neural stem cells. Quantifications are presented on right-hand panels. n = number of observed mitotic cells (late anaphase or telophase) of at least three independent experiments. The Percent deviation in staining between two cibling cells is displayed in the bottom right corner of images. Scale bars = 10μm.

In differentiation condition, we observed that mitotic mGb4 cells frequently expressed the GFAP intermediate filament. Unexpectedly, we made the observation that in paraformaldehyde-fixed cells, GFAP was asymmetrically distributed in approximately 7% of mitotic cells during the anaphase or telophase (Fig 2A). Co-staining for a spindle apparatus marker (β-tubulin) confirmed that these cells were undergoing mitosis and that the GFAP asymmetry is not simply a doublet of unrelated and attached cells (Fig 2B). The possibility existed that while GFAP filaments were present at the same level in the two daughter cells, epitopes recognized by GFAP antibodies were masked in one cell as a result of GFAP phosphorylation or because of GFAP interactions with a specific pattern in only one cell. To rule out this possibility of an IF artifact, we used two other antibodies against GFAP and this confirmed the asymmetric distribution of this intermediate filament in a small fraction of mitotic cells (Fig 2A).

As GBM are highly heterogenous tumors composed of different cell types and to exclude that GFAP could be segregated asymmetrically specifically in mGb4, we extended our observations to GBM multipotent cells derived from two other patients. We used the Gb5 culture which was previously characterized [32] and we also derived a new glioma sphere culture from a newly diagnosed GBM patient (Gb21). Gb21 cells express the typical markers for GBM multipotent cells such as CD15, CD133, Olig2 and Sox2 (S1 File). IF performed on differentiated Gb5 and Gb21 cells revealed clear events of unequal repartition of GFAP during mitosis which demonstrates the occurrence of asymmetric distribution of GFAP in independent GBM multipotent cells (S2 File).

Finally, to show that asymmetric distribution of GFAP is not restricted to human GBM cultures, we explored whether this phenomenon could also occur in non tumoral cells. For this, we used neural stem cell cultures derived from the mouse adult spinal cord [33]. These cells are cultured in the same media as glioma spheres, are highly proliferative and differentiate into astrocytes, neurons and oligodendrocytes upon growth factor removal (S3 File). Differentiation induces an increase in the level of GFAP expression concomitantly with a reduction of division rate [34]. In this condition, using IF, we observed the asymmetric repartition of GFAP in a small fraction of mitotic cells (approximately 7%) (Fig 2C). The asymmetric distribution of GFAP in multipotent cells thus appears as an evolutionary-conserved phenomenon which is not restricted to the tumoral context.

### Asymmetric distribution of GFAP-EGFP fusion protein during mitosis in mGb4 culture

**Fig 3:**
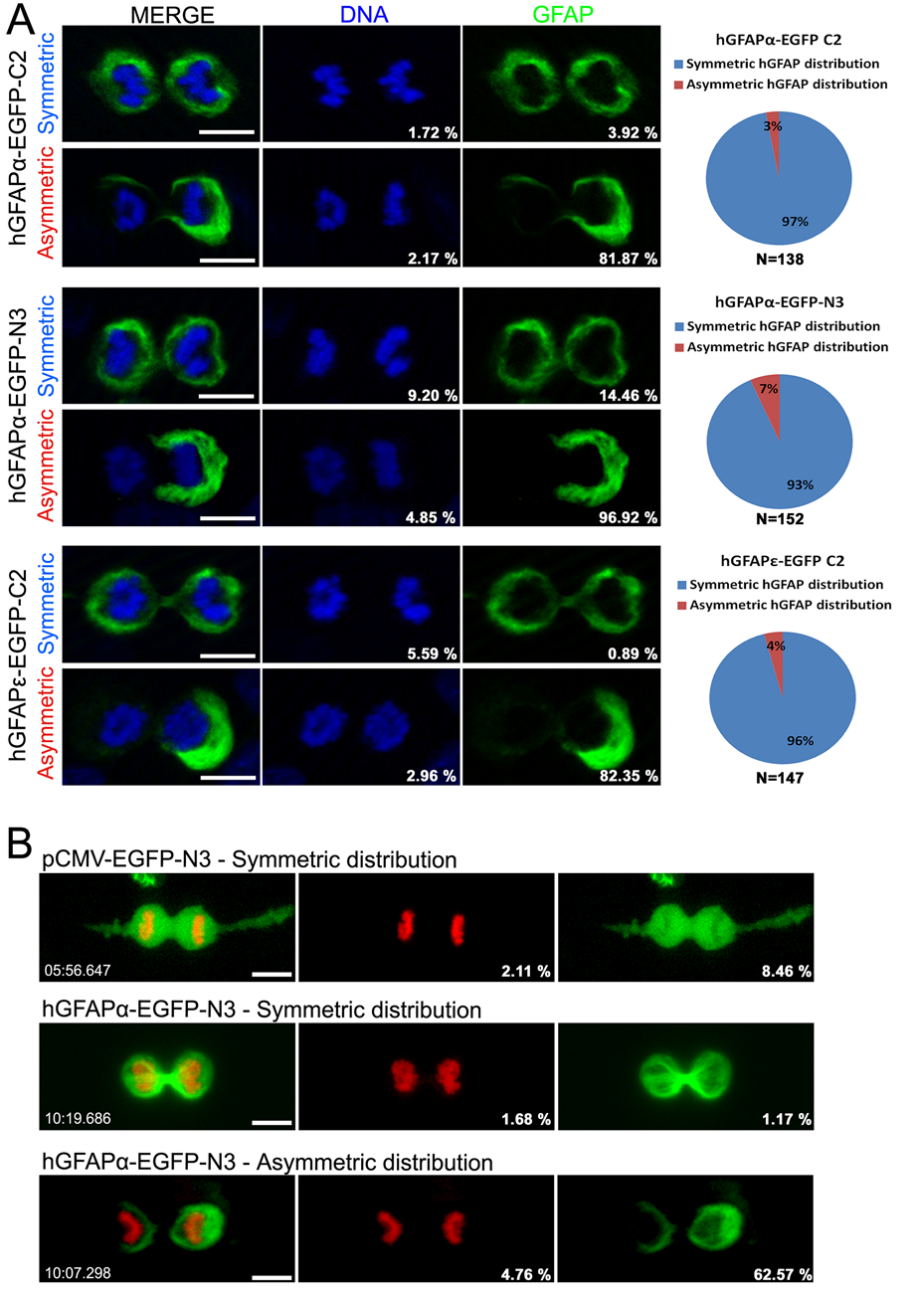
Symmetric and Asymmetric distribution of GFAP-EGFP during mitosis. **(A)** Examples of symmetric and asymmetric distribution of GFAPα-EGFP and GFAPε-EGFP in mitotic paraformaldehyde-fixed cells. For GFAPα, 2 different constructs were used in which EGFP was either fused to the C-terminal (hGFAPα-EGFP-C2) or to the N-terminal (hGFAPα-EGFP-N3). No prominent difference was observed between these 2 constructs. Quantifications are presented on the right-hand panels. n = number of observed mitotic cells (late anaphase or telophase) in three independent experiments. **(B)** Images of time-lapse confocal microscopy of mGb4 cells cotransfected with the indicated GFP constructs and H2B-mCherry (red nuclei). The pCMV-EGFP construction used as control showed only symmetric distribution of EGFP (upper panel). In contrast, cells transfected with the hGFAPα-EGFP-N3 construct showed symmetric (middle panel) or asymmetric distribution of GFAP-EGFP protein (lower panel) during mitosis. The Percent deviation in staining between two cibling cells is displayed in the bottom right corner of images. Scale bars = 10μm.

To validate further this observation using a completely independent technique, we transfected mGb4 cells with a plasmid coding for GFAP fused to EGFP. For these experiments, we used three types of constructs: the isoform alpha (GFAPα) which is the most frequent form found in the central nervous system [36, 37] and the isoform epsilon (GFAPε, also known as GFAPδ [38]) known to be preferentially expressed in astrocytes in neurogenic regions [39, 40]. For GFAPα we tested two constructs where EGFP was either fused to the N- or C-terminal of GFAP especially as it was reported that the subcellular location of fusion proteins could be influenced by the presence of EGFP [41]. After transfection of these three constructs and fixation, examination of mitotic cells confirmed an asymmetric repartition of EGFP in approximately 3–7% of the cells whatever the constructs (Fig 3A). This indicates that both isoforms of GFAP could undergo asymmetric repartition in mGb4 cells.

Finally, as an ultimate demonstration of asymmetric repartition of GFAP in GBM cells, we used confocal-videomicroscopy to directly capture the phenomenon. Fig 3B and S4, S5 and S6 Files show three examples of clear asymmetric distribution of GFAP recorded in two daughter cells undergoing cytokinesis (n = 50 observed mitosis). These films also revealed that GFAP was asymmetrically distributed in the cell several minutes before cytokinesis occurred. This indicates that the AD process leading to GFAP accumulation in one part of the cell is not concomitant to cytoplasm separation but started well ahead. As a comparison, Fig 3B and S7 File show a symmetric repartition of GFAP in the two daughter cells. We never observed asymmetric segregation of the control unfused EGFP protein (20 cells observed) (Fig 3B and S8 File) which confirms the specificity of the unequal segregation of GFAP-EGFP in a fraction of mGb4 cells.

## Discussion

Here we demonstrate that the intermediate filament GFAP can segregate asymmetrically in multipotent GBM and non tumoral neural cell division. This was also reported recently by another team [42]. The rarity (less than 10% of division) and rapidity of this event may explain why it has been unnoticed before. The low frequency of GFAP asymmetric repartition in GBM cells might have resulted from a cellular heterogeneity in the culture with cells undergoing very frequent asymmetric divisions intermingled with cells unable to do so. This possibility is however unlikely as in this study we derived and used a clonal GBM-derived cell culture. It is more probable that the observed low rate of GFAP asymmetric distribution reflects a stochastic and rare event. Alternatively, this rate could be determined by unknown complex cellular interactions favoring GFAP unequal repartition and occurring at a low frequency in the cell culture.

The function, if any, as well as the molecular machinery underlying this unequal distribution of GFAP remains to be fully addressed. The implication of a similar intermediate filament, vimentin, in the control of Golgi apparatus position, motility of mitochondria within the cell and distribution of endosomal and lysosomal protein has been reported [43]. Along this line, we hypothesized that unequal distribution of GFAP could induce asymmetric segregation of organelles in mGb4 cells. We explored this possibility by conducting double labelling for GFAP and proteins specific for the Golgi apparatus (GM130) [44], the endoplasmic reticulum (FTCD) [45] and the lysosomal compartment (AP3) [46] on mGb4 cells but no convincing co-asymmetric distributions were obtained (S9 File). A same conclusion was obtained using videomicroscopy for GFAP-GFP and a red fluorescent protein targeting the Golgi apparatus (S10 and S11 Files).

In conclusion, there are growing evidence indicating that cytoskeleton proteins are actively participating in the regulation of cell fate and asymmetric division. In fact, GFAP^**-/-**^ Vim^**-/-**^ astrocytes showed reduced endocytosis of the Notch ligand Jagged1 and displayed a reduced Notch signaling [47]. This indicates that intermediate filaments might participate in the regulation of stem cell fate by controlling major cell decision pathways such as Notch. We thus speculate that the observed asymmetric distribution of GFAP in GBM multipotent cells and neural stem cells could possibly play a role in the regulation of cell fate.

## Supporting Information

S1 File Phenotypic characterization of Gb21 GBM cells by IF and FACS. Scale bars = 1**0** μ**m.**
(TIF)

S2 File Asymmetric distribution of GFAP in Gb5 and Gb21 cells. The Percent deviation in staining between the two cibling cells is displayed in the bottom right corner of images. Scale bars = 1**0** μ**m.**
(TIF)

S3 File Differentiation of adult spinal cord neurospheres. Positive cells (arrows) for GFAP, Dcx and O4 markers are shown. Scale bars = 10 μm.
(TIF)

S4 File Videos of asymmetric distribution of GFAP-EGFP during mitosis of mGb4 cells. Scale bar = 10μm.
(MP4)

S5 File Videos of asymmetric distribution of GFAP-EGFP during mitosis of mGb4 cells. Scale bar = 10μm.
(MP4)

S6 File Videos of asymmetric distribution of GFAP-EGFP during mitosis of mGb4 cells. Scale bar = 10μm.
(MP4)

S7 File Video of symmetric distribution of GFAP-EGFP during mitosis of mGb4 cells. Scale bar = 10μm.
(MP4)

S8 File Video of symmetric distribution of EGFP during mitosis of mGb4 cells. Scale bar = 10μm.
(MP4)

S9 File **(A)** Examples of GM130 stainings (red dots) in mitotic cells with asymmetric GFAP distribution. **(B)** Examples of symmetric and asymmetric distribution of GFAP-GFP and Golgi-DsRed protein. No association between Golgi apparatus and GFAP was observed. The Percent deviation in staining between the two cibling cells is displayed in the bottom right corner of images. Scale bars = 10μm.
(TIF)

S10 File Video of symmetric distribution of GFAP-EGFP and Golgi-DsRed during mitosis of mGb4 cells. Scale bar = 10μm.
(MP4)

S11 File Video of asymmetric distribution of GFAP-EGFP and Golgi-DsRed during mitosis of mGb4 cells. Scale bar = 10μm.
(MP4)

S12 File Values for statistical analysis of asymmetry, histograms and graphs. (XLSX)

S1 Table Patient annotations. (DOCX)

S2 Table Antibody list. (DOCX)
