# Supplementary figures and images for: Asymmetric Distribution of GFAP in Glioma Multipotent Cells
Source: PLoS One. 2016 Mar 8;11(3):e0151274. doi: 10.1371/journal.pone.0151274 (PMC4783030; doi:10.1371/journal.pone.0151274)

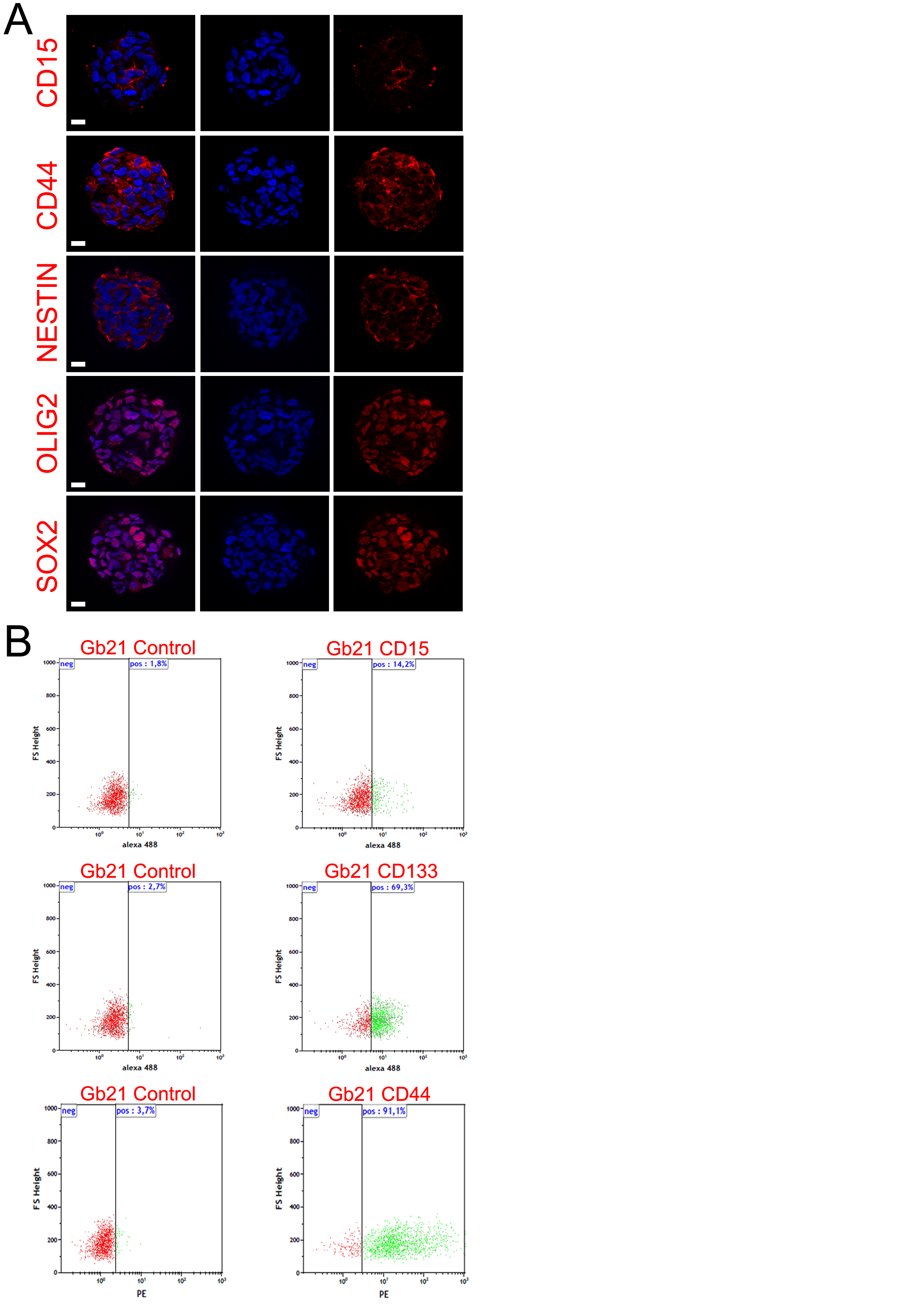

Supplement: S1 File — Scale bars = 10 μm. (TIF) [file pone.0151274.s001.tif]

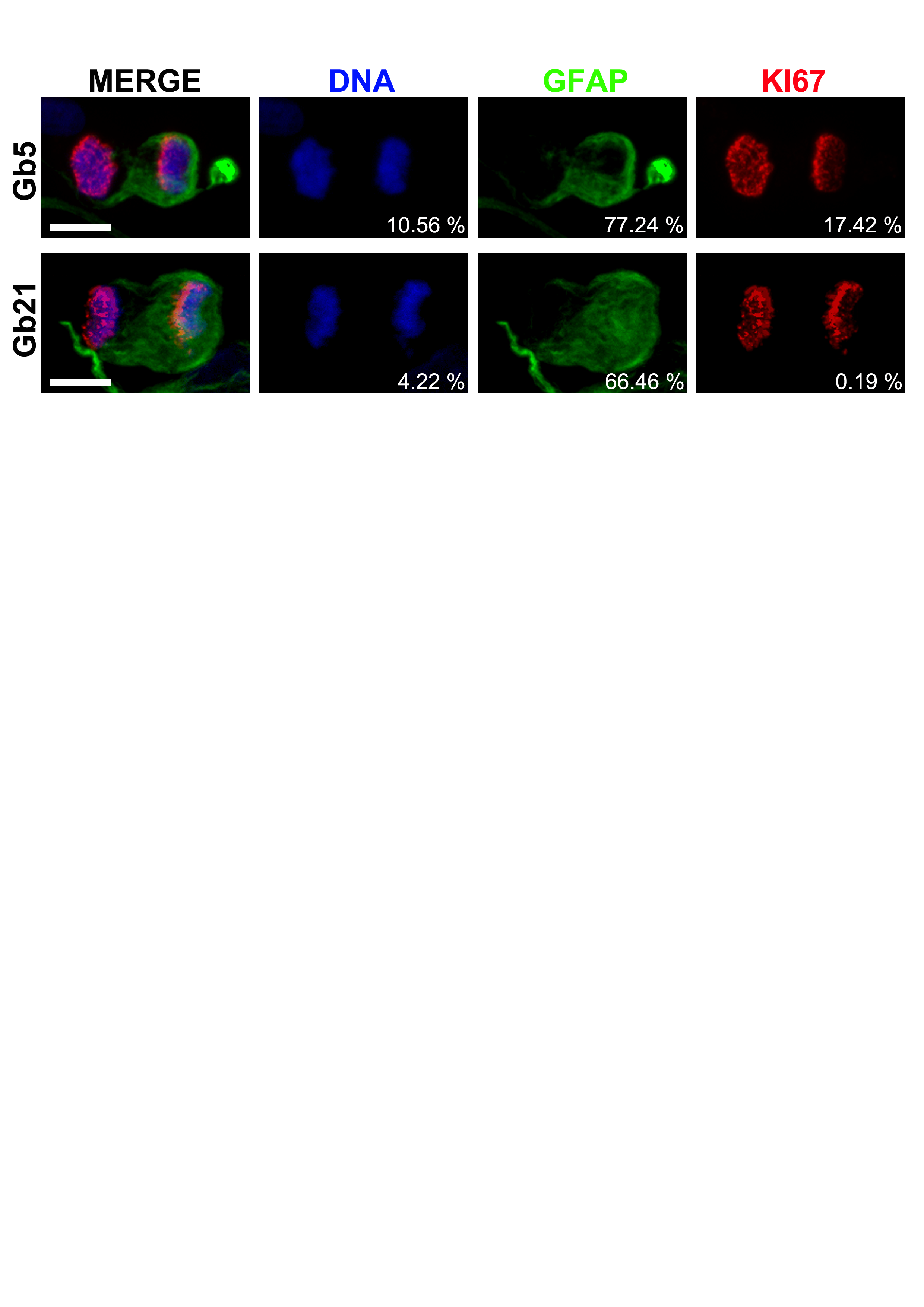

Supplement: S2 File — The Percent deviation in staining between the two cibling cells is displayed in the bottom right corner of images. Scale bars = 10 μm. (TIF) [file pone.0151274.s002.tif]

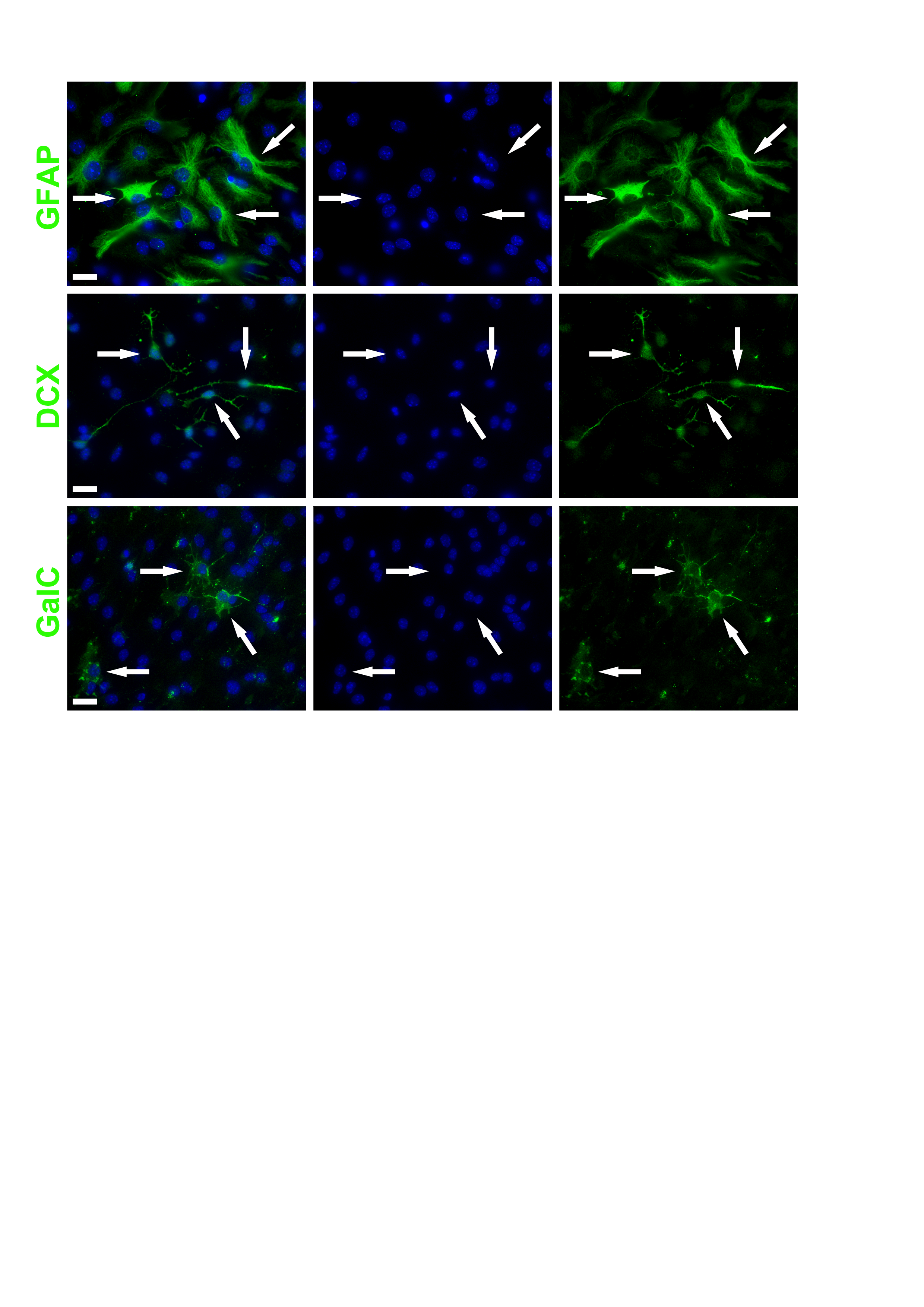

Supplement: S3 File — Positive cells (arrows) for GFAP, Dcx and O4 markers are shown. Scale bars = 10 μm. (TIF) [file pone.0151274.s003.tif]

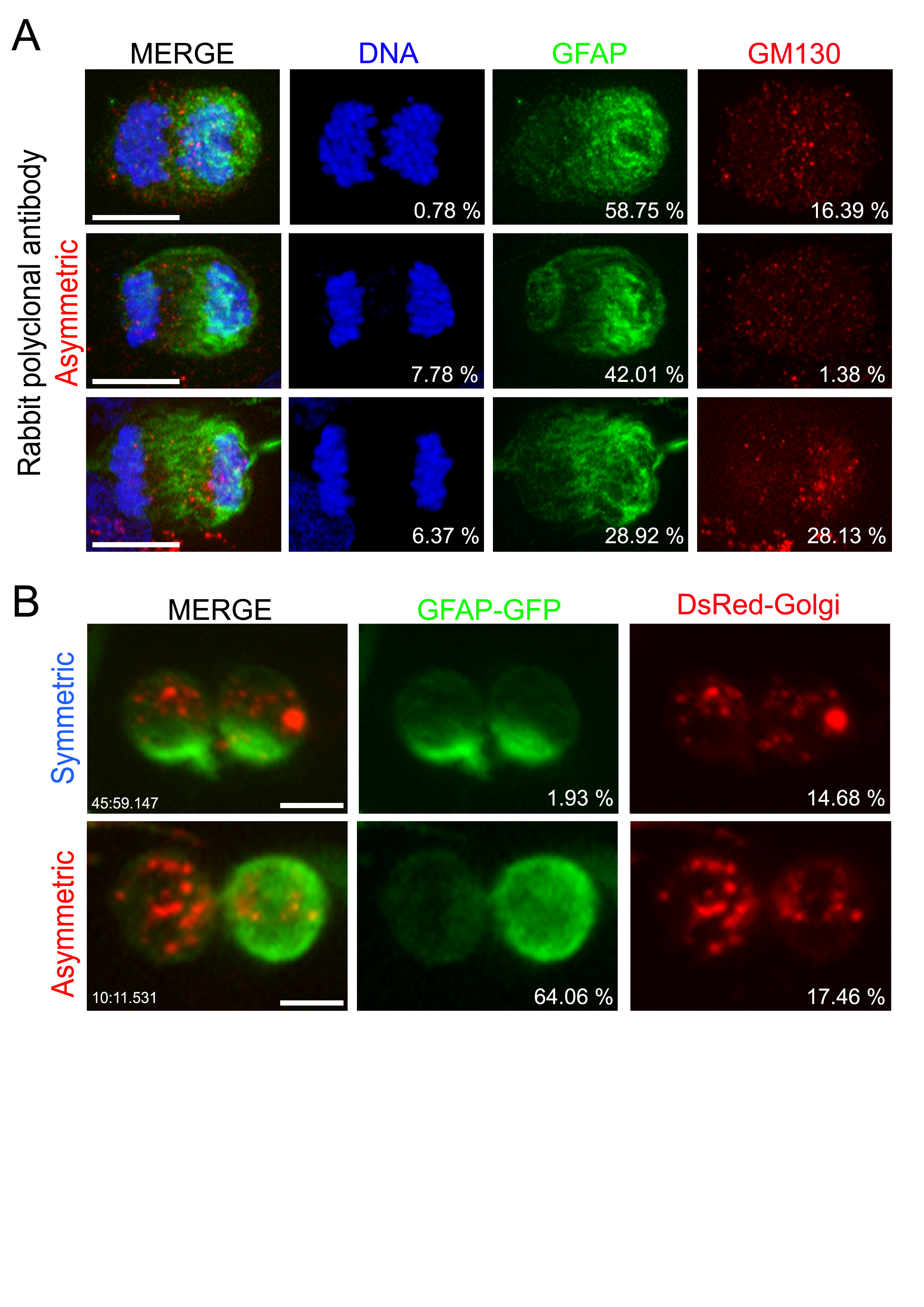

Supplement: S9 File — (A) Examples of GM130 stainings (red dots) in mitotic cells with asymmetric GFAP distribution. (B) Examples of symmetric and asymmetric distribution of GFAP-GFP and Golgi-DsRed protein. No association between Golgi apparatus and GFAP was observed. The Percent deviation in staining between the two cibling cells is displayed in the bottom right corner of images. Scale bars = 10μm. (TIF) [file pone.0151274.s009.tif]
