# Supplementary material for: Asymmetric Distribution of GFAP in Glioma Multipotent Cells
Source: PLoS One. 2016 Mar 8;11(3):e0151274. doi: 10.1371/journal.pone.0151274 (PMC4783030; doi:10.1371/journal.pone.0151274)
Supplement: S1 Table — (DOCX) [file pone.0151274.s013.docx]

**Supporting Information Table 1. Patient annotations**

| **Tumor** | **Age (years)** | **Sex** | **Topography** | **% Ki67+ cells** | **WHO grade** | **Survival (months)** |
| --- | --- | --- | --- | --- | --- | --- |
| Gb 4 | 53 | Male | Left temporal | 70 | IV | 21 |
| Gb 5 | 64 | Male | Left temporal | 30 | IV | 15 |
| Gb 21 | 62 | Female | Right Fronto-Insular | 35 | IV | Alive |
