# Supplementary material for: Asymmetric Distribution of GFAP in Glioma Multipotent Cells
Source: PLoS One. 2016 Mar 8;11(3):e0151274. doi: 10.1371/journal.pone.0151274 (PMC4783030; doi:10.1371/journal.pone.0151274)
Supplement: S2 Table — (DOCX) [file pone.0151274.s014.docx]

**Supplemental Table 1. Antibody list.**

| **Name** | **Species** | **References** | **Distributors** | **Dilution** |
| --- | --- | --- | --- | --- |
| CALPONIN | Rabbit | Ab46794 | Abcam | 1:250 |
| CD15 | Mouse | 559045 | BD Biosciences | 1:200 |
| CD44-PE | Rat | 12-0441 | Affymetrix ebiosciences | 1:200 |
| CD133 | Mouse | 130-090-422 | Miltenyi Biotec | 1:5 |
| DCX | Rabbit | Ab18723 | Abcam | 1:5000 |
| GalC | Mouse | MAB342 | Millipore | 1:200 |
| GFAP | Rabbit | z0334 | Dako | 1:5000 |
| GFAP | Mouse | G3893 | Sigma | 1:500 |
| GFAP | Chicken | Ab4674 | Abcam | 1:2000 |
| HuNu | Mouse | MAB1281 | Millipore | 1 :500 |
| Ki67 | Mouse | 5560003 | BD Pharmingen | 1 :500 |
| MAP2ab | Mouse | M4403 | Sigma | 1:500 |
| OLIG2 | Rabbit | 18953 | IBL | 1:500 |
| SOX2 | Goat | Sc-17320 | Santa cruz | 1:200 |
| TUBB3 | Mouse | T8660 | Sigma | 1:500 |
